# Relationship between changes in resting-state spontaneous brain activity and cognitive impairment in patients with CADASIL

**DOI:** 10.1186/s10194-019-0982-3

**Published:** 2019-04-17

**Authors:** Jingjing Su, Mengxing Wang, Shiyu Ban, Liang Wang, Xin Cheng, Fengchun Hua, Yuping Tang, Houguang Zhou, Yu Zhai, Xiaoxia Du, Jianren Liu

**Affiliations:** 1 Department of Neurology and Jiuyuan Municipal Stroke Center, Shanghai Ninth People’s Hospital, Shanghai Jiao Tong University School of Medicine, 639 Zhizaoju Road, Shanghai, 200011 People’s Republic of China; 2 Shanghai Key Laboratory of Magnetic Resonance and Department of Physics, School of Physics and Materials Science, East China Normal University, 3663 North Zhongshan Road, Shanghai, 200062 People’s Republic of China; 3 College of Medical Imaging, Shanghai University of Medicine & Health Sciences, 279 Zhouzhu Highway, Shanghai, 201318 People’s Republic of China; 4 Department of Neurology, Huashan Hospital, Fudan University, 12 Middle Wulumuqi Road, Shanghai, 200040 People’s Republic of China; 5 PET Center, Huashan Hospital, Fudan University, 518 East Wuzhong Road, Shanghai, 200235 People’s Republic of China; 6 Department of Geriatrics Neurology, Huashan Hospital, Fudan University, 12 Middle Wulumuqi Road, Shanghai, 200040 People’s Republic of China

**Keywords:** Amplitude of low-frequency fluctuation, CADASIL, Cognitive impairment, Resting-state fMRI

## Abstract

**Background:**

Cerebral autosomal dominant arteriopathy with subcortical infarcts and leukoencephalopathy (CADASIL) mainly manifests with cognitive impairment. Cognitive deficits in patients with CADASIL are correlated with structural brain changes such as lacunar lesion burden, normalized brain volume, and anterior thalamic radiation lesions, but changes in resting-state functional brain activity in patients with CADASIL have not been reported.

**Methods:**

This study used resting-state functional magnetic resonance imaging (fMRI) to measure the amplitude of low-frequency fluctuation (ALFF) in 22 patients with CADASIL and 44 healthy matched controls. A seed-based functional connectivity (FC) analysis was used to investigate whether the dysfunctional areas identified by ALFF analysis exhibited abnormal FC with other brain areas. Pearson’s correlation analysis was used to detect correlations between the ALFF z-score of abnormal brain areas and clinical scores in patients with CADASIL.

**Results:**

Patients with CADASIL exhibited significantly lower ALFF values in the right precuneus and cuneus (Pcu/CU) and higher ALFF values in the bilateral superior frontal gyrus (SFG) and left cerebellar anterior and posterior lobes compared with controls. Patients with CADASIL showed weaker FC between the areas with abnormal ALFF (using peaks in the left and right SFG and the right Pcu/CU) and other brain areas. Importantly, the ALFF z-scores for the left and right SFG were negatively associated with cognitive performance, including Mini-Mental State Examination (MMSE) and Montreal Cognitive Assessment scores (MoCA), respectively, whereas those of the right Pcu/CU were positively correlated with the MMSE score.

**Conclusions:**

This preliminary study provides evidence for changes in ALFF of the right Pcu/CU, bilateral SFG and left cerebellar anterior and posterior lobes, and associations between ALFF values for abnormal brain areas and cognitive performance in patients with CADASIL. Therefore, spontaneous brain activity may be a novel imaging biomarker of cognitive impairment in this population.

## Background

Cerebral autosomal dominant arteriopathy with subcortical infarcts and leukoencephalopathy (CADASIL) has been recognized as the most common heritable cause of recurrent stroke and vascular dementia. This disease is caused by pathogenic mutations in the NOTCH3 gene on chromosome 19, which encodes a transmembrane receptor primarily expressed in vascular smooth muscle cells and pericytes of the small penetrating cerebral and leptomeningeal arteries [1]. Typical pathological changes of CADASIL include accumulation of a non-amyloid granular osmiophilic material and the NOTCH3 protein in the vessel wall. The majority of patients with CADASIL have a family history in the absence of vascular disease risk factors such as hypertension, diabetes, coronary heart disease, and smoking. The essential clinical manifestations of CADASIL include migraine with aura, subcortical ischemic events, mood disturbances, and cognitive impairment. It has been reported that 80% of patients with this disorder have cognitive deficits, principally in the domains of executive function, information processing speed, attention, and memory [1, 2].

Structural cerebral magnetic resonance imaging (MRI) in patients with CADASIL reveals age-dependent widespread white matter lesions, seen as large symmetrical hyperintensities on T2-weighted and fluid-attenuated inversion recovery (FLAIR) imaging in periventricular areas and in the centrum semiovale [3]. Furthermore, lacunar infarcts and microbleeds are frequently visible in the basal ganglia and brainstem [4, 5]. Studies employing diffusion tensor imaging (DTI), a quantitative measure of white matter integrity, have revealed extensive brain microstructural changes in patients with CADASIL [6], with different cognitive functions being correlated with white matter structural integrity at different sites [7]. Furthermore, other conventional MRI parameters, such as lacunar lesion burden and brain atrophy, have important impacts on cognitive function and have become essential predictors of cognitive decline in patients with CADASIL [8–13]. One study indicated that reaction time on a simple task may serve as a marker of early cognitive changes in patients with CADASIL [14]. Reaction time is negatively associated with corpus callosum area, determined on three-dimensional T1 MRI sequences using a validated methodology [15]. Collectively, these results demonstrate that MRI measures of brain structures have close associations with cognitive functioning in patients with CADASIL.

Resting-state functional magnetic resonance imaging (fMRI) has been widely used to investigate changes in global functional network connectivity and local spontaneous neuronal activity in the brain at rest. Cognitive functioning, including executive function and processing speed, depend on the integrity of the frontal–subcortical and frontoparietal networks [16–18]. A preliminary study by Cullen et al. demonstrated that intrinsic frontoparietal functional networks are also detectable in patients with CADASIL and are associated with cognitive measures, particularly measures of executive ability and speed [19]. Although these abnormalities in functional network connectivity can be used to reveal the comprehensive and integrative characteristics of brain areas, that study was unable to assess the abnormalities in regional neuronal activity. The amplitude of low-frequency fluctuation (ALFF) is a method that has been used to evaluate local spontaneous neuronal activity in a resting-state fMRI analysis based on blood oxygenation level dependent (BOLD) signals. It reflects the amplitude of neuronal activity within a single voxel by calculating the square root of the power spectrum in a frequency range (typically 0.01–0.08 Hz) [20]. ALFF is effective for examining neuropsychiatric disease-related neuronal activity within specific brain regions [21]. Previous studies have shown the specific patterns of ALFF in mild cognitive impairment (MCI), Alzheimer’s disease (AD) and subcortical ischemic vascular dementia (SIVD) and these alterations in ALFF have revealed significant correlations with cognitive performance in these patients [22–24]. Measurement of ALFF may aid in early diagnosis and illness monitoring of AD and may become a sensitive marker of AD pathology such as Aβ/pTau ratio regardless of clinical diagnosis, which can provide novel insights into the pathophysiological mechanisms of AD [25–27]. Thus, we assumed that brain functional activity is more likely to be altered in these patients in the resting-state based on the aforementioned abnormalities in cognitive behavior and brain structure in patients with CADASIL [1–5]. However, whether there are changes in ALFF values and whether these aberrant changes have impacts on cognitive impairment in patients with CADASIL remain poorly understood. In this study, we used ALFF analysis to investigate regional spontaneous neuronal activity in patients with CADASIL by resting-state fMRI and examined their correlations with cognitive performance.

## Methods

### Participants

Twenty-two genetically confirmed patients with CADASIL were recruited from 11 families with CADASIL referred to the Department of Neurology at Shanghai Ninth People’s Hospital between May 2015 and July 2017. The proband for each family was selected based on recurrent stroke and leukoencephalopathy, and each was eventually diagnosed genetically with CADASIL. Subsequently, each family member underwent genetic screening to search for other cases in the group and those with a genetic diagnosis of CADASIL were included in this study. Among these cases, 4 patients had not any clinical symptoms and others manifested stroke, headache and memory impairment. Patients with severe untreated depression or anxiety or neurodegenerative disorders were excluded. Forty-four healthy controls of comparable age and sex were also enrolled for comparison. All the controls did not experience stroke, headache and cognitive impairment and their family members had not cerebrovascular diseases, headache or cerebrovascular risk factors. All neurological and psychiatric diseases were excluded based on both clinical examinations and MRI structured interviews including lacunae and white matter lesions for controls.

### Clinical assessment

Clinical information including family history, age at first symptoms, history of previous stroke or transient ischemic attack (TIA), headache and cerebrovascular disease risk factors such as hypertension, diabetes, coronary heart disease, hyperlipidemia and smoking was recorded. Neurological deficits were assessed using the National Institute of Health Stroke Scale (NIHSS) and the modified Rankin scale (mRs) as assessments of disability.

The neuropsychological assessment was carried out by a clinical neuropsychologist. Cognitive scores including the Mini-Mental State Examination (MMSE) and Montreal Cognitive Assessment (MoCA) were recorded. State anxiety and depression were evaluated using the Hamilton Anxiety Scale (HAMA) and the Hamilton Depression Scale (HAMD), respectively.

### MRI acquisition

Structural MRI and fMRI scans were performed at East China Normal University on a 3.0 Tesla Siemens Trio Tim system using a 12-channel head coil. All subjects’ head movements were minimized with custom-fit foam pads. The structural MRI scan included T1- and T2-weighted and FLAIR imaging. The FLAIR sequence parameters were repetition time, 9000 ms; echo time, 93 ms; field of view, 199 × 220 mm^2^; number of slices, 30; and slice thickness, 3.5 mm. The resting-state fMRI images were acquired using a T2*-weighted gradient-echo echo-planar imaging pulse sequence with the following parameters: repetition time, 2000 ms; echo time, 30 ms; flip angle, 90°; field of view, 220 × 220 mm^2^; matrix size, 64 × 64; number of slices, 33; slice thickness, 3.5 mm; and a total of 210 volumes. The whole-brain anatomical volume was obtained using a high-resolution T1-weighted three-dimensional magnetization-prepared rapid-acquisition gradient-echo pulse sequence with the following parameters: repetition time, 2530 ms; echo time; 2.34 ms; field of view, 256 × 256 mm^2^; number of slices, 192; slice thickness, 1 mm; and flip angle, 7°.

### Fazekas scale score

White matter hyperintensities (WMHs) were shown on T2-weighted or FLAIR sequences. The degree of WMH severity was assessed visually on axial FLAIR images using the modified Fazekas scale [28, 29], the most widely used scale to describe WMH severity. The scale divides WMHs into periventricular and deep WMHs. Periventricular WMHs were graded according to the following patterns: 0 = absent; 1 = caps or pencil-thin lining; 2 = smooth halo; and 3 = irregular WMHs extending into deep white matter. Deep WMHs were graded according to the following patterns: 0 = absent or single punctate foci; 1 = multiple punctate foci; 2 = beginning confluence of foci; and 3 = large fused foci. Total scores were acquired by adding the periventricular and deep WMH scores, and the results were compared using quantitative analyses.

### Resting-state fMRI data preprocessing

The resting-state fMRI data were preprocessed with Data Processing and Analysis of Brain Imaging (DPABI) software on a personal computer. DPABI is a newly developed toolbox which is loaded into statistical parametric mapping software (SPM12; http://www.fil.ion.ucl.ac.uk/spm/software/spm12), and is opened by MATLAB software (Math Works, Natick, MA, USA). It is designed to make data analysis require fewer manual operations. The first 10 volumes were discarded to avoid signal instability. The preprocessing steps included a slice-timing correction, realignment of the functional data to each participant’s first images, and co-registration of the functional to structural images. The sessions were spatially normalized to the standard Montreal Neurological Institute space. Gaussian spatial smoothing [6 mm, full-width at half-maximum (FWHM)] was performed on the functional images. Finally, subjects with head movement > 2 mm were excluded from the analysis, and the signals from cerebral white matter and cerebrospinal fluid were also removed using a general linear model.

## Data analysis

### ALFF analysis

The ALFF analysis was based on previous preprocessed results [20]. The time sequences for a given voxel were transformed to the frequency series by fast Fourier transform, and the square root of the power spectrum was calculated and filtered across 0.01–0.08 Hz. The average square root was considered to be the ALFF value. To reduce individual differences among subjects, the average ALFF value was subtracted from the ALFF value of each voxel and then divided by the standard deviation of the whole-brain ALFF map to obtain the standard ALFF value.

### Functional connectivity (FC)

A seed-based FC analysis was used to investigate whether the dysfunctional areas shown in the ALFF results exhibited abnormal FC with other brain areas. The brain areas with significant differences in the ALFF analysis were the seed regions. The mean time series of the seed regions was correlated with the time series of each voxel in the whole brain to obtain FC maps, which were converted to z-score maps by Fisher’s z-transformation to improve the normality of the data distribution.

### Correlational analysis

Individual mean ALFF z-scores and z-transformed FC (zFC) values for the surviving CADASIL clusters were extracted for a Pearson’s correlation analysis using the clinical data, including the Fazekas, MMSE, MoCA, NIHSS, and mRs scores. Furthermore, according to the different cognitive domains in MMSE and MoCA, a subanalysis was employed to detect a more specific correlation with the mean ALFF z-scores for the surviving clusters of CADASIL. Significant correlations were determined based on *p*-values less than 0.05.

## Statistical analysis

The maps of the significant differences in ALFF of the 22 CADASIL and the 44 controls were compared using voxel-wise two-sample *t* tests with age and gender as covariates. Additionally, the FC maps used voxe-wise two-sample *t* tests with age and gender as covariates within a brain mask. To address the issue of multiple comparisons, the ALFF and FC statistical maps were assigned thresholds at *p* < 0.001 (voxel level), and family-wise errors (FWE) were corrected to *p* < 0.05 at the cluster level. The surviving clusters were reported.

Pearson’s correlation analysis was used to detect correlations with clinical scores in patients. Demographic data between two groups were compared using Pearson’s chi-squared test for gender and independent-sample *t* tests for age, MMSE, MoCA, HAMD and HAMA. Significant differences were determined based on *p* < 0.05.

## Results

### Demographic and clinical data of the CADASIL and control groups

The demographic and clinical data of the CADASIL and control groups are presented in Table 1. Age, gender, and median anxiety and depression symptom scores did not differ between the CADASIL and control groups. However, the cognitive scores showed significant differences between two groups. The main clinical manifestations of patients included subcortical ischemic events, headache and cognitive impairment.

**Table 1:** Demographic and clinical characteristics of the CADASIL and control groups

	CADASIL group (*n* = 22)	Control group (*n* = 44)	*p-*values
Male/Female	13/9	26/18	1
Age at visit (years)	49.0 ± 14.2	48.5 ± 13.7	0.873
Family history, n (%)	19 (86.4)	-	-
Age at first symptom (years)	45.7 ± 13.3	-	-
Previous stroke or TIA (times)	1.7 ± 2.7	-	-
Vascular disease risk factors, n (%)	10 (45.5)	-	-
Headache, n (%)	3 (13.6)	-	-
Fazekas score	2.1 ± 1.2	-	-
MMSE	23.3 ± 6.3	28.6 ± 1.1	0.001
MoCA	20.7 ± 7.9	27.9 ± 1.4	0.000
HAMD	7.0 ± 7.1	4.0 ± 1.8	0.067
HAMA	5.9 ± 6.0	3.7 ± 1.6	0.108
NIHSS	1.2 ± 1.9	-	-
mRs	1.3 ± 1.2	-	-

Values are mean ± standard deviation (SD) or numbers and percentages. *CADASIL* cerebral autosomal dominant arteriopathy with subcortical infarcts and leukoencephalopathy, *MMSE* Mini-Mental State Examination, *MoCA* Montreal Cognitive Assessment, *HAMD* Hamilton Depression Scale, *HAMA* Hamilton Anxiety Scale, *NIHSS* National Institute of Health Stroke *Scale*, mRs modified Rankin scale, *SD* standard deviation, *TIA* transient ischemic attack

### ALFF

**Fig. 1:**
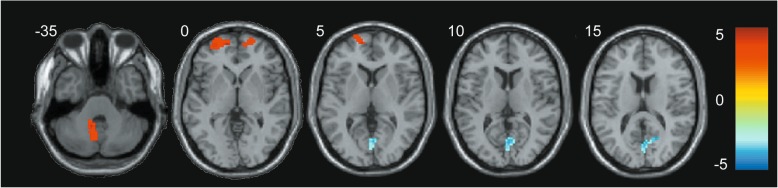
Significant differences in ALFF between the CADASIL and control groups. The CADASIL group showed significantly lower ALFF values in the right Pcu/CU (in blue), and higher ALFF values in the bilateral SFG and left cerebellar anterior and posterior lobes (in red) compared with controls. The color-coded scale was referred to the t-values. ALFF: amplitude of low-frequency fluctuation; CADASIL: cerebral autosomal dominant arteriopathy with subcortical infarcts and leukoencephalopathy; Pcu/CU: precuneus and cuneus; SFG: superior frontal gyrus

Patients with CADASIL had significantly lower ALFF values in the right precuneus and cuneus (Pcu/CU) compared with controls (Fig. 1; Table 2). The ALFF values in the bilateral superior frontal gyrus (SFG) and left cerebellar anterior and posterior lobes were increased markedly in the patients with CADASIL compared with the values for controls (Fig. 1; Table 2).

**Table 2:** Significant inter-group differences in ALFF among CADASIL and controls

Predominant regions in cluster	Cluster size	Peak *T* value	MNI coordinates			Cluster level *P*_FWE-corr_
			x	y	z	
ALFF reduction in CADASIL						
Right Pcu/CU (BA 7/BA 17)	210	−5.31	6	− 81	30	0.000
ALFF increase in CADASIL						
Left SFG (BA 10)	88	5.28	−12	60	3	0.008
Right SFG (BA 10)	62	4.82	21	60	− 6	0.034
Left cerebellar anterior and posterior lobes	58	4.38	−9	− 51	− 36	0.044

The surviving clusters of ALFF were assigned thresholds of *p* < 0.001 and FWE-corrected to *p* < 0.05 at the cluster level

*ALFF* amplitude of low-frequency fluctuation, *CADASIL* cerebral autosomal dominant arteriopathy with subcortical infarcts and leukoencephalopathy, *Pcu/CU* precuneus and cuneus, *SFG* superior frontal gyrus, *BA* Brodmann’s area, *MNI* Montreal Neurological Institute

### Seed-based FC

**Fig. 2:**
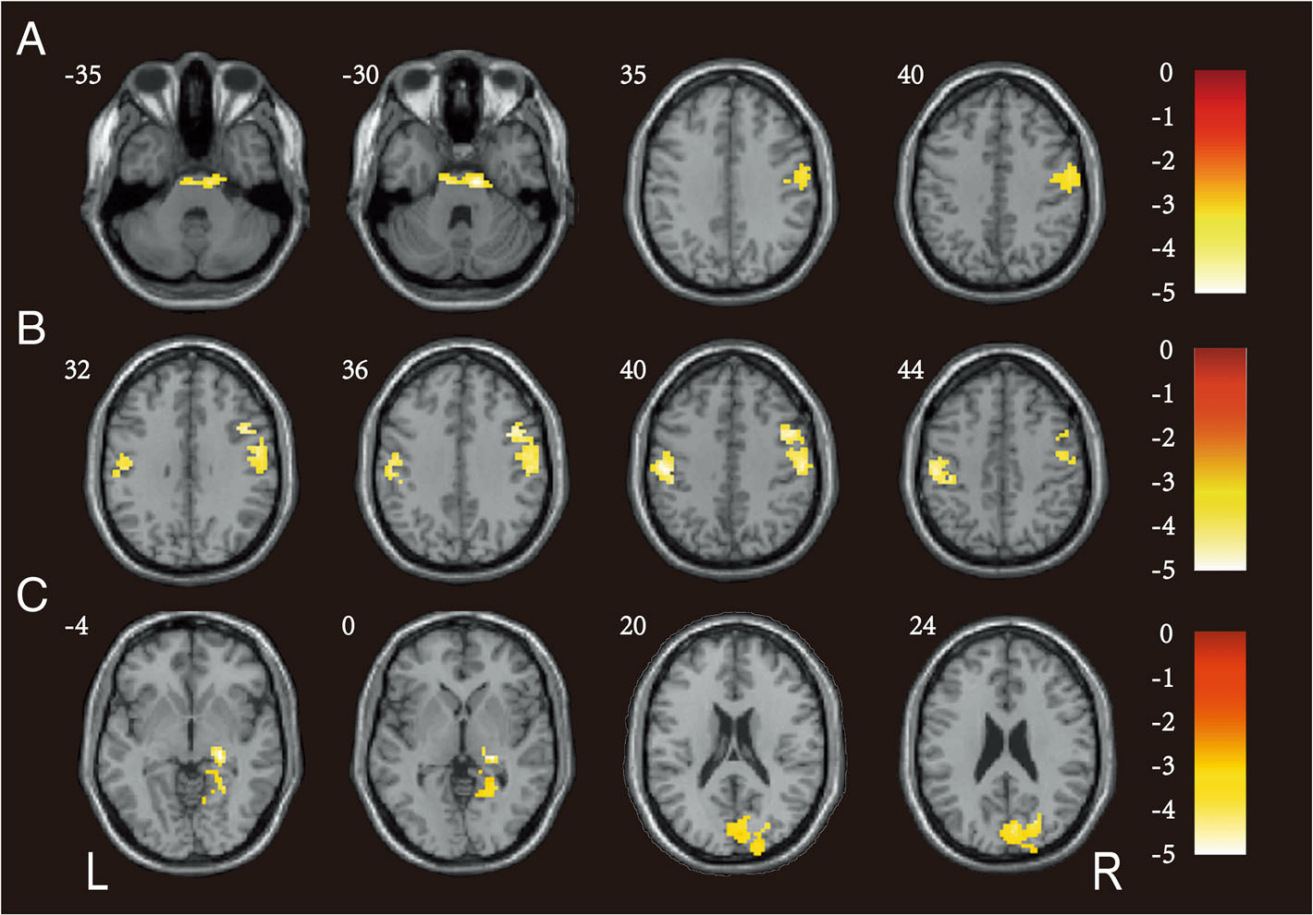
FC of brain areas with abnormal ALFF values was compared with that in other brain areas between the CADASIL and control groups. **a** The CADASIL group had weaker FC between the left SFG and right precentral gyrus and between the left SFG and bilateral pons. **b** Weaker FC between the right SFG and right precentral gyrus and between the right SFG and left postcentral gyrus was observed in the CADASIL group compared to the control group. **c** The patients with CADASIL displayed significantly weaker FC between the right Pcu/CU and right CU and between the right Pcu/CU and right PHG. The color-coded scale was referred to the t-values. FC: functional connectivity; ALFF: amplitude of low-frequency fluctuation; CADASIL: cerebral autosomal dominant arteriopathy with subcortical infarcts and leukoencephalopathy; SFG: superior frontal gyrus; Pcu/CU: precuneus and cuneus; CU: cuneus; PHG: parahippocampal gyrus

To further investigate FC between the areas of abnormal ALFF and other brain areas, we compared the FC of the bilateral SFG, right Pcu/CU and left cerebellar anterior and posterior lobes with other brain regions in the CADASIL and control groups. The patients with CADASIL showed weaker FC between the left SFG and right precentral gyrus and between the left SFG and bilateral pons compared with controls (Fig. 2a; Table 3). Similarly, weaker FC between the right SFG and right precentral gyrus and between the right SFG and left postcentral gyrus was observed in the patients with CADASIL compared to the controls (Fig. 2b; Table 3). The results from the peak in the right Pcu/CU as the region of interest seed showed weaker FC between the right Pcu/CU and right CU and between the right Pcu/CU and right parahippocampal gyrus (PHG) in patients with CADASIL compared to controls (Fig. 2c; Table 3). However, no abnormal FC was found between left cerebellar anterior and posterior lobes and other brain regions in CADASIL compared to controls (Table 3).

**Table 3:** Significant inter-group differences in the FC analysis of patients with CADASIL and controls

Predominant regions in cluster	Cluster size	Peak *T* value	MNI coordinates			Cluster level *P*_FWE-corr_
			x	y	z	
CADASIL < controls (seed region: left SFG)						
Right precentral gyrus	116	−3.78	60	− 6	36	0.037
Bilateral pons	115	−4.79	12	− 18	− 33	0.038
CADASIL < controls (seed region: right SFG)						
Right precentral gyrus	239	−4.14	48	9	39	0.001
Left postcentral gyrus	133	− 4.48	− 51	− 21	39	0.020
CADASIL < controls (seed region: right Pcu/CU)						
Right cuneus	239	−5.42	6	−87	36	0.002
Right PHG	184	− 5.35	24	−27	−3	0.007
CADASIL compared to controls (seed region: left cerebellar anterior and posterior lobes)						
Right pon	109	−4.93	9	−18	− 33	0.057
Left cerebrum	31	−3.84	−24	−15	− 3	0.519

The surviving clusters of FC were assigned thresholds at *p* < 0.001 and FWE-

corrected to *p* < 0.05 at the cluster level

*FC* functional connectivity, *CADASIL* cerebral autosomal dominant arteriopathy with subcortical infarcts and leukoencephalopathy, *SFG* superior frontal gyrus, *Pcu/CU* precuneus and cuneus, *PHG* parahippocampal gyrus, *MNI* Montreal Neurological Institute

### Correlations with clinical scores

**Fig. 3:**
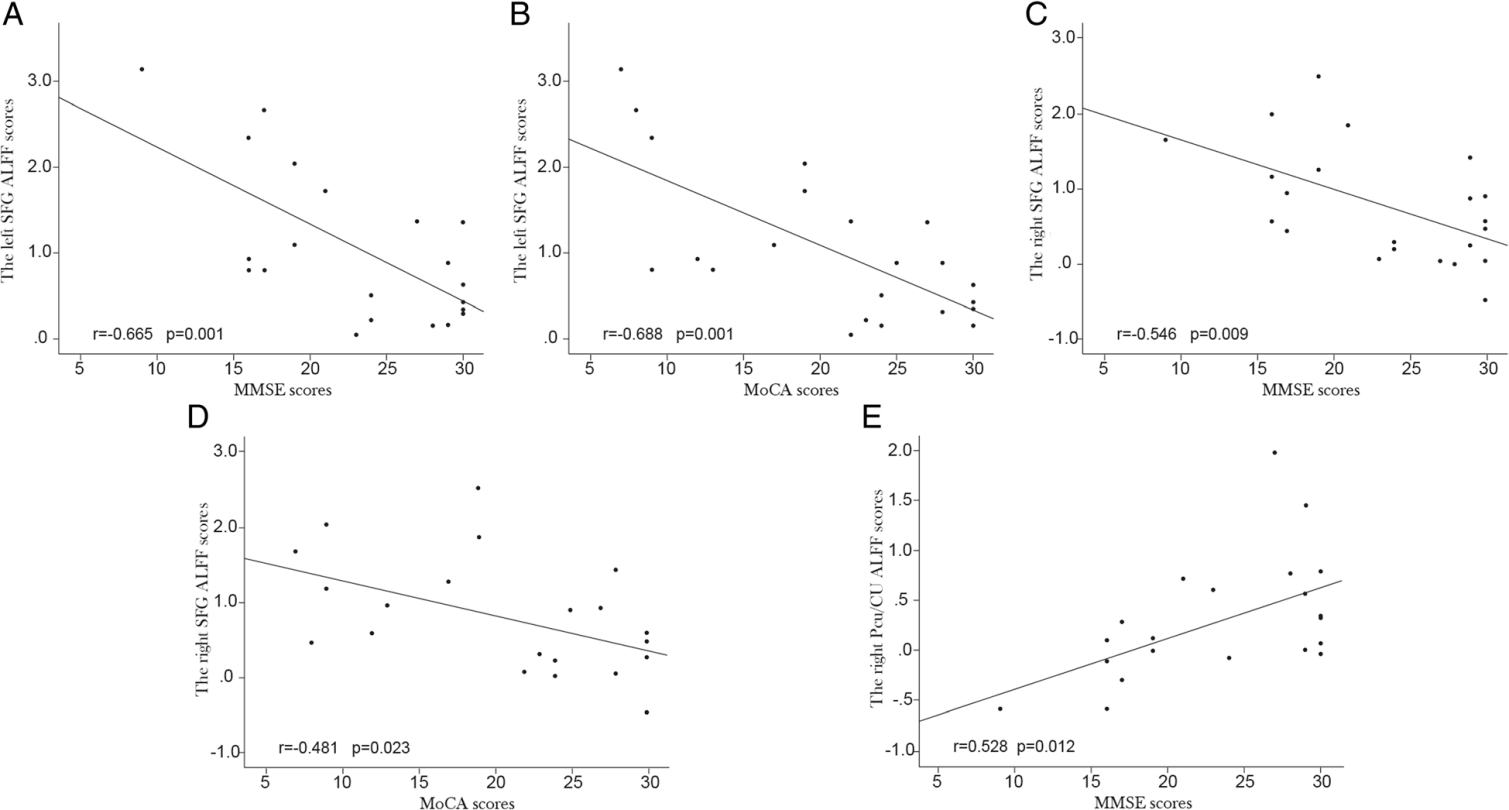
Significant correlations between the individual mean ALFF z-scores and cognitive measures for the surviving clusters in the CADASIL group. **a** The left SFG and MMSE; *r* = − 0.665, *n* = 22, *p* = 0.001. **b** The left SFG and MoCA; *r* = − 0.688, *n* = 22, *p* = 0.000. **c** The right SFG and MMSE; *r* = − 0.546, *n* = 22, *p* = 0.009. **d** The right SFG and MoCA; *r* = − 0.481, *n* = 22, *p* = 0.023. **e** The right Pcu/CU and MMSE; *r* = 0.528, *n* = 22, *p* = 0.012. ALFF: amplitude of low-frequency fluctuation; CADASIL: cerebral autosomal dominant arteriopathy with subcortical infarcts and leukoencephalopathy; SFG: superior frontal gyrus; MMSE: Mini-Mental State Examination; MoCA: Montreal Cognitive Assessment; Pcu/CU: precuneus and cuneus

The ALFF z-scores in the brain areas where ALFF was increased, including the left and right SFG, were negatively associated with cognitive performance, including scores on the MMSE (*r* = − 0.665, *p* = 0.001; *r* = − 0.546, *p* = 0.009, respectively) and MoCA (*r* = − 0.688, *p* = 0.000; *r* = − 0.481, *p* = 0.023, respectively) (Fig. 3a, b, c and d). However, the ALFF z-scores for the right Pcu/CU, which showed a decreased ALFF value, were positively correlated with the MMSE scores (*r* = 0.528, *p* = 0.012) (Fig. 3e). Furthermore, ALFF z-scores of the left SFG (*r* = 0.429, *p* = 0.046) and the left cerebellar anterior and posterior lobes (*r* = 0.432, *p* = 0.045) were positively correlated with Fazekas scores, and ALFF z-scores of the left and right SFG (*r* = 0.487, *p* = 0.021; *r* = 0.453, *p* = 0.034, respectively) were positively correlated with mRs scores. No correlation was found between the mean ALFF z-score in the significantly altered brain regions and the NIHSS scores.

According to the different cognitive domains in MMSE and MoCA, the ALFF z-scores for the right SFG were negatively correlated with the orientation and working memory scores in MMSE (*r* = − 0.486, *p* = 0.025; *r* = − 0.513, *p* = 0.017, respectively). The negative associations had also been found between the ALFF values of the left cerebellar anterior and posterior lobes and the cognitive scores for the orientation and working memory in MMSE (*r* = − 0.447, *p* = 0.042; *r* = − 0.451, *p* = 0.040, respectively) and MoCA (*r* = − 0.537, *p* = 0.012; *r* = − 0.455, *p* = 0.038, respectively). Moreover, the ALFF z-scores for the left cerebellar anterior and posterior lobes were negatively correlated with attention and executive function scores in MoCA (*r* = − 0.438, *p* = 0.047; *r* = − 0.725, *p* = 0.000, respectively).

The zFC values of the right precentral gyrus were positively correlated with MMSE scores (*r* = 0.449, *p* = 0.036). There were no significant correlations between the mean zFC values within the clusters and the Fazekas, MoCA, NIHSS, and mRs scores in CADASIL.

## Discussion

CADASIL highly resembles sporadic SIVD. However, an ideal experimental model of sporadic SIVD has been absent until now. CADASIL could serve as a model for studying the relationship between cerebrovascular pathology and cognitive impairment in patients with sporadic SIVD and thereby become a model of pure vascular dementia [1, 2]. Therefore, research about the mechanisms underlying CADASIL will help in early recognition and prevention of CADASIL and sporadic SIVD.

This is the first study to investigate the aberrant changes in intrinsic, spontaneous brain activity in patients with CADASIL by measuring ALFF values from resting-state fMRI signals. The ALFF values revealed abnormal brain activity in the right Pcu/CU, bilateral SFG and left cerebellar anterior and posterior lobes, weak FC between the areas with abnormal ALFF and other brain areas, and close associations between changes in ALFF values and cognitive performance.

In this study, a pronounced decease in ALFF in the right Pcu/CU was observed in patients with CADASIL. The Pcu and CU are located in the medial parietal and inferior occipital lobes, respectively [30, 31]. Three distinct FC patterns have been demonstrated in the Pcu based on resting- state fMRI data. The anterior Pcu is functionally connected with the sensorimotor region, the central Pcu with the cognitive/associative region, and the posterior Pcu with adjacent visual cortical regions [30]. Therefore, the Pcu has been considered the hub of the frontoparietal central executive network, and it is thus a vulnerable brain region [32]. The Pcu is often activated during retrieval of episodic memory, and this activation is related to visual imagery during memory processes [33]. Furthermore, some findings have suggested that Pcu volume is associated with impaired anterograde memory due to posterior cortical atrophy, which is driven by well-recognized deficits in visual imagery processing [34]. The Pcu and CU are also involved in several distinct domains, including cognitive reserve, self-awareness of memory, and strategic learning [31]. In this study, the decreased ALFF values in the right Pcu/CU suggest that patients with CADASIL might have the impaired Pcu/CU cognitive functions described above and that Pcu/CU dysfunction might be involved in the pathogenic mechanisms underlying CADASIL. Here, the patterns of functional change in the Pcu/CU of patients with CADASIL were partially consistent with patients with SIVD, MCI or AD [22, 23, 35, 36]. Studies using ^18^F-fluorodeoxyglucose positron emission tomography in patients with CADASIL have shown hypometabolism in the Pcu/CU [37]. In addition, Craggs et al. used post-mortem brains from patients with CADASIL to observe severe vessel sclerosis in these two brain regions [38]. Similar results, such as cortical thinning, hypometabolism, hypoperfusion, and loss of synapses in the Pcu/CU, have also been reported in patients with AD by structural MRI, fMRI, and electron microscopy [39–41]. These findings may account for the low resting-state activation in the Pcu/CU of patients with CADASIL in the present study.

We also observed an increase in ALFF values in the bilateral SFG and left cerebellar anterior and posterior lobes in the patients with CADASIL. The SFG brain region belongs to Brodmann’s area 10 (BA 10), which is the largest frontal brain region and is known as the anterior prefrontal cortex (PFC), frontopolar prefrontal cortex, or rostral prefrontal cortex [42]. BA 10 has been implicated in multiple integrative roles, including working memory, episodic memory retrieval, abstract reasoning, decision making, behavior control, monitoring of internal states, odor evaluation, nociceptive processing, and perceptual metacognition [42–44]. The cytoarchitecture of BA10 is characterized by more dendritic spines, a higher spine density, and a lower cell body density compared with other areas of the cortex, suggesting that BA 10 is more likely to accomplish these tasks of information integration [45]. In contrast, the cerebellum includes the cerebellar anterior and posterior lobes. Anatomical, neuroimaging, clinical, and behavioral studies have demonstrated that the cerebellum is engaged in the motor domain as well as higher-level cognitive and affective functions. It has been established that sensorimotor tasks activate the anterior lobe, whereas the posterior lobe is involved in higher-level tasks, including language and verbal working memory, executive functions, spatial tasks, and emotional processing. Therefore, a “sensorimotor” and a “cognitive” cerebellum has been proposed [46–48]. In particular, language and executive tasks activate the posterior lobe and are involved in prefrontal–cerebellar loops [46]. Taken together, the present data show an increase of ALFF activities in the bilateral SFG (BA 10) and left cerebellar anterior and posterior lobes in patients with CADASIL. Therefore, we inferred that the higher spontaneous activity in BA 10 and the cerebellum might be compensatory mechanisms for cognitive deficits in these patients, acting to enhance their functional connectivity. Similarly, increased brain activities in the SFG and posterior lobe of the cerebellum have also been reported in patients with AD and SIVD [22, 49].

In the present study, negative associations were found between ALFF values for the bilateral SFG (BA 10) and cognitive performance, including MMSE and MoCA scores, in patients with CADASIL. Conversely, there was a positive association between the ALFF value for the right Pcu/CU and MMSE scores in these patients. These results indicate that patients with greater global cognitive impairment may exhibit more obvious compensation for ALFF activity in the bilateral BA 10, while similar protective effects were not found in the Pcu/CU. Compensatory reactions in spontaneous brain activity have also been detected in patients with SIVD [22, 49]. According to the different cognitive domains in MMSE and MoCA, we also found negative associations between ALFF values for the right BA 10 and left cerebellar anterior and posterior lobes and orientation and working memory scores, and between ALFF values for the left cerebellar anterior and posterior lobes and attention and executive function scores. These results indicated that resting-state ALFF analysis may be predictive of cognitive deficits, particularly in the domains of orientation, working memory, attention and executive function in CADASIL. In our study, we also found positive associations of the ALFF values of the left BA 10 and left cerebellar anterior and posterior lobes with Fazekas scores and of ALFF values of the bilateral BA 10 with mRs scores, indicating that increased BA 10 and cerebellum brain activity could aid in detecting illness severity in patients with CADASIL.

It has been widely accepted that CADASIL is a subcortical disease with infarcts and leukoencephalopathy. However, postmortem neuropathological examinations and transcranial MRI have shown that patients with CADASIL have widespread neuronal apoptosis in the cerebral cortex, predominantly in layers 3 and 5, and cholinergic dysfunction in the motor cortex [50, 51]. These cortical deficits are related to subcortical infarcts and white matter lesions underlying the primary motor cortex. Therefore, CADASIL has been considered a disease of disconnection due to disruption of either the cortico–cortical or cortical–subcortical networks in the white matter of the frontal lobe, which may explain the executive dysfunction and motor deficits in patients with CADASIL [38, 52]. Recent evidence from resting-state FC highlights the connections between BA 10 and multiple cognitive networks, including the default mode network (DMN), the executive control network, the salience network, and the sensorimotor network. The DMN includes the medial PFC (mainly BA 10), the Pcu, the posterior cingulate cortex (PCC), and the PHG, and plays a vital role in internal process monitoring and memory retrieval [42]. Furthermore, DTI data have offered novel anatomical evidence for large BA 10 fibers contributing to the human cortico–ponto–cerebellar system in the pontine nuclei, indicating that BA 10 may play a vital role in increasing the connections between the cortico–cortical or cortical–subcortical networks by participating in the motor system [53]. Although weaker FC was observed between BA 10 and the major sensorimotor cortex, including the precentral gyrus and postcentral gyrus, and between BA 10 and the subcortical pons in the present study, we considered them as insufficient compensation. Therefore, we proposed the protective mechanism of BA 10 and the sensorimotor areas counteracting the disconnection between the cortical and subcortical networks in patients with CADASIL. Here, decreased FC between the Pcu/CU and CU and between the Pcu/CU and PHG was also detected, consistent with the aberrant connectivity in the occipital–parietal lobe in patients with MCI and AD [54]. Furthermore, we inferred that the dysfunction in the DMN, including BA 10, Pcu, PCC, and PHG, might be implicated in the pathological mechanism underlying CADASIL.

Some limitations of this study should be discussed. First, the relatively small sample of patients with CADASIL reduced the power to detect significant effects. Further study will be necessary with a larger sample size. Second, we did not classify the patients according to the degree of illness. In the future, we will observe functional changes in early- and late-stage patients. Third, a brief screening of cognitive function, including the MMSE and MoCA, was used in this study. Future studies will focus on more detailed cognitive domains, such as executive function, processing speed, attention, and working memory.

## Conclusions

This study revealed spontaneous aberrant ALFF values and correlations of these values with cognitive functions in patients with CADASIL. It is well known that resting-state fMRI is a quick and repeatable method without radiation injury, and it has therefore been used to measure features of brain function for early detection and illness monitoring. Therefore, spontaneous brain activity may have the potential to become a predictor of cognitive status in patients with CADASIL. These findings will help to elucidate the causal mechanisms underlying CADASIL and to develop therapeutic strategies.

## Abbreviations

ALFF: Amplitude of low-frequency fluctuation
CADASIL: Cerebral autosomal dominant arteriopathy with subcortical infarcts and leukoencephalopathy
DTI: Diffusion tensor imaging
FC: Functional connectivity
fMRI: functional magnetic resonance imaging
HAMA: Hamilton Anxiety Scale
HAMD: Hamilton Depression Scale
MMSE: Mini-Mental State Examination
MoCA: Montreal Cognitive Assessment scores
mRs: modified Rankin scale
NIHSS: National Institute of Health Stroke Scale
Pcu/CU: Precuneus and cuneus
SFG: Superior frontal gyrus

## References

[1] Chabriat H, Joutel A, Dichgans M (2009). Cadasil. Lancet Neurol.

[2] Di Donato I, Bianchi S, De Stefano N (2017). Cerebral autosomal dominant Arteriopathy with subcortical infarcts and leukoencephalopathy (CADASIL) as a model of small vessel disease: update on clinical, diagnostic, and management aspects. BMC Med.

[3] Singhal S, Rich P, Markus HS (2005). The spatial distribution of MR imaging abnormalities in cerebral autosomal dominant arteriopathy with subcortical infarcts and leukoencephalopathy and their relationship to age and clinical features. AJNR Am J Neuroradiol.

[4] Hervé D, Godin O, Dufouil C (2009). Three-dimensional MRI analysis of individual volume of lacunes in CADASIL. Stroke.

[5] Dichgans M, Holtmannspötter M, Herzog J (2002). Cerebral microbleeds in CADASIL: a gradient-echo magnetic resonance imaging and autopsy study. Stroke.

[6] Mascalchi M, Pantoni L, Giannelli M (2017). Diffusion tensor imaging to map brain microstructural changes in CADASIL. J Neuroimaging.

[7] O’Sullivan M, Barrick TR, Morris RG et al (2005) Damage within a network of white matter regions underlies executive dysfunction in CADASIL. Neurology 65:1584–1590. 10.1212/01.wnl.0000184480.07394.fb 10.1212/01.wnl.0000184480.07394.fb 16301485

[8] Duering M, Zieren N, Hervé D (2011). Strategic role of frontal white matter tracts in vascular cognitive impairment: a voxel-based lesion-symptom mapping study in CADASIL. Brain.

[9] Viswanathan A, Gschwendtner A, Guichard JP et al (2007) Lacunar lesions are independently associated with disability and cognitive impairment in CADASIL. Neurology 69:172–179. 10.1212/01.wnl.0000265221.05610.70 10.1212/01.wnl.0000265221.05610.70 17620550

[10] Lee JS, Choi JC, Kang SY (2011). Effects of lacunar infarctions on cognitive impairment in patients with cerebral autosomal- dominant arteriopathy with subcortical infarcts and leukoencephalopathy. J Clin Neurol.

[11] Liem MK, van der Grond J, Haan J (2007). Lacunar infarcts are the main correlate with cognitive dysfunction in CADASIL. Stroke.

[12] Liem MK, Lesnik Oberstein SA, Haan J (2009). MRI correlates of cognitive decline in CADASIL: a 7-year follow-up study. Neurology.

[13] Peters N, Holtmannspötter M, Opherk C (2006). Brain volume changes in CADASIL: a serial MRI study in pure subcortical ischemic vascular disease. Neurology.

[14] Jouvent E, Reyes S, De Guio F (2015). Reaction time is a marker of early cognitive and behavioral alterations in pure cerebral small vessel disease. J Alzheimers Dis.

[15] Delorme S, De Guio F, Reyes S (2017). Reaction time is negatively associated with Corpus callosum area in the early stages of CADASIL. AJNR Am J Neuroradiol.

[16] Duering M, Gonik M, Malik R (2013). Identification of a strategic brain network underlying processing speed deficits in vascular cognitive impairment. Neuroimage.

[17] Petersen SE, Posner MI (2012). The attention system of the human brain: 20 years after. Annu Rev Neurosci.

[18] Stuss DT (2006). Frontal lobes and attention: processes and networks, fractionation and integration. J Int Neuropsychol Soc.

[19] Cullen B, Moreton FC, Stringer MS (2016). Resting state connectivity and cognitive performance in adults with cerebral autosomal-dominant arteriopathy with subcortical infarcts and leukoencephalopathy. J Cereb Blood Flow Metab.

[20] Zang YF, He Y, Zhu CZ (2007). Altered baseline brain activity in children with ADHD revealed by resting-state functional MRI. Brain Dev.

[21] Xiao F, Wang T, Gao L et al (2018) Frequency-dependent changes of the resting BOLD signals predicts cognitive deficits in asymptomatic carotid artery stenosis. Front Neurosci 12:416. 10.3389/fnins.2018.00416 10.3389/fnins.2018.00416 PMC6021536 29977187

[22] Liu C, Li C, Yin X (2014). Abnormal intrinsic brain activity patterns in patients with subcortical ischemic vascular dementia. PLoS One.

[23] Ni L, Liu R, Yin Z (2016). Aberrant spontaneous brain activity in patients with mild cognitive impairment and concomitant lacunar infarction: a resting-state functional MRI study. J Alzheimers Dis.

[24] Liu X, Wang S, Zhang X (2014). Abnormal amplitude of low-frequency fluctuations of intrinsic brain activity in Alzheimer’s disease. J Alzheimers Dis.

[25] Liang P, Xiang J, Liang H (2014). Altered amplitude of low-frequency fluctuations in early and late mild cognitive impairment and Alzheimer’s disease. Curr Alzheimer Res.

[26] Yang L, Yan Y, Wang Y (2018). Gradual Disturbances of the Amplitude of Low-Frequency Fluctuations (ALFF) and Fractional ALFF in Alzheimer Spectrum. Front Neurosci.

[27] Ren P, Lo RY, Chapman BP (2016). Longitudinal alteration of intrinsic brain activity in the striatum in mild cognitive impairment. J Alzheimers Dis.

[28] Fazekas F, Chawluk JB, Alavi A (1987). MR signal abnormalities at 1.5 T in Alzheimer’s dementia and normal aging. AJR Am J Roentgenol.

[29] Boutet C, Rouffiange-Leclair L, Schneider F (2016). Visual assessment of age-related white matter Hyperintensities using FLAIR images at 3 T: inter- and intra-rater agreement. Neurodegener Dis.

[30] Margulies DS, Vincent JL, Kelly C (2009). Precuneus shares intrinsic functional architecture in humans and monkeys. Proc Natl Acad Sci U S A.

[31] Park S, Ryu SH, Yoo Y (2018). Neural predictors of cognitive improvement by multi-strategic memory training based on metamemory in older adults with subjective memory complaints. Sci Rep.

[32] Menon V (2011). Large-scale brain networks and psychopathology: a unifying triple network model. Trends Cogn Sci.

[33] Delazer M, Ischebeck A, Domahs F (2005). Learning by strategies and learning by drill--evidence from an fMRI study. Neuroimage.

[34] Ahmed S, Irish M, Loane C (2018). Association between precuneus volume and autobiographical memory impairment in posterior cortical atrophy: beyond the visual syndrome. Neuroimage Clin.

[35] Li C, Liu C, Yin X (2014). Frequency-dependent changes in the amplitude of low-frequency fluctuations in subcortical ischemic vascular disease (SIVD): a resting-state fMRI study. Behav Brain Res.

[36] Cha J, Hwang JM, Jo HJ (2015). Assessment of functional characteristics of amnestic mild cognitive impairment and Alzheimer's disease using various methods of resting-state FMRI analysis. Biomed Res Int.

[37] Tatsch K, Koch W, Linke R (2003). Cortical hypometabolism and crossed cerebellar diaschisis suggest subcortically induced disconnection in CADASIL: an 18F-FDG PET study. J Nucl Med.

[38] Craggs LJ, Yamamoto Y, Ihara M (2014). White matter pathology and disconnection in the frontal lobe in cerebral autosomal dominant arteriopathy with subcortical infarcts and leukoencephalopathy (CADASIL). Neuropathol Appl Neurobiol.

[39] Lajoie I, Nugent S, Debacker C (2017). Application of calibrated fMRI in Alzheimer's disease. Neuroimage Clin.

[40] Niskanen E, Könönen M, Määttä S (2011). New insights into Alzheimer's disease progression: a combined TMS and structural MRI study. PLoS One.

[41] Scheff SW, Price DA, Schmitt FA (2013). Synapse stability in the precuneus early in the progression of Alzheimer’s disease. J Alzheimers Dis.

[42] Peng K, Steele SC, Becerra L (2018). Brodmann area 10: collating, integrating and high level processing of nociception and pain. Prog Neurobiol.

[43] Babiloni C, Vecchio F, Bares M (2008). Functional coupling between anterior prefrontal cortex (BA10) and hand muscle contraction during intentional and imitative motor acts. Neuroimage.

[44] Fleming SM, Ryu J, Golfinos JG (2014). Domain-specific impairment in metacognitive accuracy following anterior prefrontal lesions. Brain.

[45] Ramnani N, Owen AM (2004). Anterior prefrontal cortex: insights into function from anatomy and neuroimaging. Nat Rev Neurosci.

[46] Stoodley CJ, Schmahmann JD (2009). Functional topography in the human cerebellum: a meta-analysis of neuroimaging studies. Neuroimage.

[47] Wagner MJ, Kim TH, Savall J (2017). Cerebellar granule cells encode the expectation of reward. Nature.

[48] Buckner RL (2013). The cerebellum and cognitive function: 25 years of insight from anatomy and neuroimaging. Neuron.

[49] Diciotti S, Orsolini S, Salvadori E (2017). Resting state fMRI regional homogeneity correlates with cognition measures in subcortical vascular cognitive impairment. J Neurol Sci.

[50] Viswanathan A, Gray F, Bousser MG (2006). Cortical l neuronal apoptosis in CADASIL. Stroke.

[51] Manganelli F, Ragno M, Cacchiò G (2008). Motor cortex cholinergic dysfunction in CADASIL: a transcranial magnetic demonstration. Clin Neurophysiol.

[52] Duering M, Righart R, Csanadi E (2012). Incident subcortical infarcts induce focal thinning in connected cortical regions. Neurology.

[53] Ramnani N, Behrens TE, Johansen-Berg H (2006). The evolution of prefrontal inputs to the cortico-pontine system: diffusion imaging evidence from Macaque monkeys and humans. Cereb Cortex.

[54] Li Q, Wu X, Xie F (2018). Aberrant connectivity in mild cognitive impairment and Alzheimer disease revealed by multimodal neuroimaging data. Neurodegener Dis.

